# Facial Neuralgia

**Published:** 1904-12-15

**Authors:** Jas. E. Powers


### FACIAL NEURALGIA.

BY DR. JAS. E. POWERS.

On February 2, 1904, Miss A. B. was referred to me for treatment. I found her suffering from severe pain all over the right side of the head, including the ear, eye, and back of the neck. I proceeded to make a careful examination of the mouth, face, etc., and found the motion of the mouth very much impaired. It was with the greatest effort and most severe pain that she succeeded in opening the mouth wide enough for me to insert my finger to make examination.

I found the inside of the mouth apparently healthy; all the teeth of the maxilla on the affected side were present. On the mandible the teeth were also present with the exception of the first and third molars. Pressure caused by my fingers rubbed lightly over the space formerly occupied by the third molar would cause almost intolerable pain.

I now proceeded to obtain the following history: In the autumn of 1898 she suffered much pain from an abscessed third molar, accompanied by swelling, etc. After a time the tooth was extracted, it breaking several times in various attempts to dislodge it—the patient insisted that it was at least six times. Severe pain resulted which lasted for several weeks, becoming less and less, but which did not entirely disappear until after the operation subsequently described was performed.

In December, 1899, severe pain in the ear, eye and face caused her physician to fear that another abscess was forming, and so it proved. After a few days it broke on the lingual surface of the jaw, discharging a peculiar dark-colored liquid into the mouth. Another abscess now formed on the jaw, in the region of the first molar, and was incised on the external surface. The patient was now unable to perform her duties for two months, a delay which caused the physician to conclude that the abscesses were due to poverty of the blood. A few months later the pain which had been present ever since 1898—varying in severity of course—became almost unbearable, and a dentist was consulted, who decided that the first molar was the cause of all the trouble, and extracted it. A dull pain continued, and a physician was called, who said that in time she would have decay of the bone in this region.

During the winter of 1902 and 1903 the diagnosis made by the physician was neuralgia of the bone, which continued until the beginning of this year.

After obtaining the above history, and making an examination, I decided that the cause was somewhere in the region of the third molar, thinking that the trouble experienced in removing this tooth might suggest—First, a small piece of root remaining; second, an injury to the bone; third, a sharp edge of roughened bone causing pressure on the inferior dental nerve, inducing irritation along this branch and by reflex action affecting the ophthalmic and superior maxillary branches of the nerve; then by its relation to the sympathetic nervous system affecting the whole side of the head.

Operation was advised, and the patient consented. It consisted of the following procedures: I made a crucial incision from the ramus to the second molar, dissected back the tissue, and exposed the surface of the bone, which was very rough. I now passed my probe backward along the border of the bone; and it entered a canal near the angle which seemed to extend to the inferior dental canal. I curetted the bone until all the rough surface was made smooth. I next smoothed the inner surface of the canal described above, and washed thoroughly with a solution of glycothymoline, hydrogen dioxid and warm water. The nurse irrigated this thoroughly every three hours, and packed the surgical opening with cotton saturated with a solution of campho-phenique and orthoform.

There was a small amount of pus present each day for three days after operation, but from the fourth day on there was none. The pain was very severe the day following the operation, and I prescribed antikamnia and codein five-grain tablets, two every two hours for two days. The pain decreased each day after the third day until the twenty-third day, when she was discharged, cured. The pain has now entirely disappeared, and three months have elapsed with no recurrence.

The canal referred to above is not the inferior dental canal, but one probably made by the operator in extracting the tooth.

There was no root remaining, and the trouble was due to a spiculum of bone causing pressure, as before stated.—*Cosmos.*

